# Carotid Body Size on CT Angiography in Patients With COVID-19 Pneumonia

**DOI:** 10.3389/bjbs.2025.14489

**Published:** 2025-05-27

**Authors:** Kamber Goksu, Ahmet Vural, Ahmet Nedim Kahraman

**Affiliations:** Department of Radiology, University of Health Sciences Fatih Sultan Mehmet Training and Research Hospital, Istanbul, Türkiye

**Keywords:** carotid body, COVID-19, pneumonia, computed tomography angiography, sympathetic nervous system

## Abstract

**Purpose:**

Many pathophysiological theories have been expressed regarding increased sympathetic activity along with respiratory failure in patients with COVID-19 pneumonia. In addition, the carotid bodies, which are directly related to increased blood oxygen levels and sympathetic activity, are known to be very rich in the angiotensin-converting enzyme 2 (ACE2) receptor, which the COVID-19 causative virus uses to enter the cell. Therefore, the probability of carotid bodies being affected in patients with COVID-19 pneumonia is quite high. Carotid bodies can be visualized with contrast-enhanced CT angiography (CTA), and we aimed to visualize possible carotid body enlargement in COVID-19 patients with CTA.

**Methods:**

We retrospectively evaluated patients who were hospitalized for COVID-19 pneumonia during the pandemic in our hospital and who had CTA examinations at least 3 months after treatment. We drew a Region of Interest (ROI) from the periphery of both carotid bodies and measured the area from the widest part. Similarly, measurements were taken in the control group without a history of COVID-19, and the results of the two groups were compared statistically.

**Results:**

We performed measurements on CTA images of 104 control subjects and 108 patients. The total carotid body area of the patients with COVID-19 pneumonia was 4.9 ± 3.7 mm^2^, and the carotid body area of the control group was 3.7 ± 2.4 mm^2^. In comparing the two groups, the carotid body area was found to be statistically significantly larger (p < 0.05) in patients with COVID-19 pneumonia.

**Conclusion:**

The size of the carotid body was found to be larger in patients with COVID-19 pneumonia compared to the control group. This finding may indicate conditions that lead to the activation of carotid body chemo and baroreceptors, such as increased sympathetic activity and a decrease in blood oxygen pressure in patients with COVID-19 pneumonia. Apart from this, it may also be possible for the carotid body to be directly infected with the virus. More specific studies that shed light on this aspect are needed.

## Introduction

Since the beginning of 2020, there have been over 650 million recorded cases of the coronavirus disease 2019 (COVID-19) epidemic worldwide [1]. The viral culprit severe acute respiratory syndrome coronavirus 2 (SARS-CoV-2) causes clinical manifestations that often involve multiple organs, most typically the respiratory, cardiovascular, and neurological systems. Symptoms have been shown to persist for weeks or even months, leading to long-term consequences. Viral-induced damage to the lung often results in hypoxemia, and its long-term persistence may be linked to reduced physiological adaptation to hypoxia [2]. Studies on the (long-term) course of SARS-CoV-2 infection have been published, international guidelines have been written and are constantly updated [3].

Long COVID, also referred to as “post-acute sequelae of COVID-19,” is a multisystemic condition consisting of usually severe symptoms following respiratory system infection caused by severe acute respiratory syndrome coronavirus 2 (SARS-CoV-2). With an estimated incidence of approximately 10% of people infected, at least 65 million people are thought to have symptoms of long COVID, based on more than 650 million documented cases of COVID-19 worldwide [1]. This number is likely much higher due to many undocumented cases. The incidence is estimated to be 10%–30% in non-hospitalized cases, 50%–70% in hospitalized cases [4, 5], and 10%–12% in vaccinated cases [6, 7]. Long COVID is seen at all ages and is thought to be related to the severity of the disease in the acute phase [8]. Following SARS-CoV-2 infection, many patients experience dozens of symptoms in multiple organ systems. Long COVID encompasses many symptoms associated with common disorders such as cardiovascular, thrombotic and cerebrovascular disease, type 2 diabetes, myalgic encephalomyelitis/chronic fatigue syndrome (ME/CFS), and dysautonomia, especially postural orthostatic tachycardia syndrome (POTS) [9]. Symptoms may persist for years, especially in new-onset cases of ME/CFS, and dysautonomia can be expected to persist throughout life [8]. It is reported that a significant portion of individuals with Long COVID cannot return to work, contributes to a serious labor shortage [10]. There is currently no approved, effective treatment. Therefore, it is important to identify possible mechanisms that may cause long COVID symptoms.

The carotid body (CB) is the primary chemosensory organ. It is sensitive to hypoxemia, increased arterial carbon dioxide, changes in pH, hypoglycemia, and changes in blood flow [11]. CBs are located at the level of the carotid artery bifurcation, on the inferomedial side, and in the periadventitial tissue [11]. In animal and human cadaver studies, it has been reported that CB hypertrophy (physical size or weight) can be seen in the presence of various chronic diseases such as hypertension, congestive heart failure, asthma, chronic obstructive pulmonary disease, and cirrhosis. All of these diseases are characterized by abnormal sympathetic activity [12–17].

Diseases such as hypertension, chronic lung disease, kidney diseases, congestive heart failure (CHF), diabetes mellitus, and obesity are associated with increased sympathetic nerve activity [18, 19]. COVID-19 infection, on the other hand, may increase sympathetic discharge through emotional distress, changes in blood gases, immune/inflammatory factors, or angiotensin converting enzyme (ACE)1/ACE2 imbalance, apart from the comorbidities listed above [20–22]. In either case, increased sympathetic activity in COVID-19 patients can be classified as short or long-term “post-COVID-19″Dysautonomia (DSN) [21]. Post-COVID-19 DSN is a poorly understood aspect of the COVID-19 pandemic as it can often overlap with clinical features and autonomic and motor-sensory symptoms [23].

It is known that ACE2 has an important regulatory role in the Renin-angiotensin system (RAS). It has been reported that the severe acute respiratory syndrome coronavirus 2 (SARS-CoV-2) virus functionally uses ACE2 receptors in the human body [24]. CB may be a potential target for COVID-19 because it is part of the local RAS and contains ACE2 receptors. Therefore, although sufficient studies have not yet been conducted, it is thought that SARS-CoV-2 may cause CB infection [25]. The intense expression of ACE2 in the carotid body parenchyma suggests that O2-sensing glomus cells may be potential targets for SARS-CoV-2 infection. Growing evidence indicates that SARS-CoV-2 circulates in the blood and can infect the lung epithelium as well as other ACE2-expressing tissues, such as the olfactory neuroepithelium, the cardiovascular system, and the gastrointestinal tract, causing multiple and distinct functional changes [26]. Therefore, based on the high expression of ACE2 found in human CB, it is possible that SARS-CoV-2 infection of chemosensory glomus cells may alter the ability of these cells to detect changes in arterial O2 pressure, resulting in failure to recognize hypoxemia. Data reveal high individual variability of ACE2 expression in human CB tissue; this may explain the difference in response to hypoxia in COVID-19 patients [25]. It is thought that SARS-CoV-2 infection may cause biochemical changes in glomus cells by selectively altering mitochondrial O2 sensing mechanisms at an early stage [24]. In more advanced stages, SARS-CoV-2 infection can lead to inflammation and glomus cell death, thereby reducing the amount of chemosensitivity elements in the CB and thus the ability to respond to hypoxemia [25].

When evaluated together with this information, it can be thought that possible increased sympathetic activity in COVID-19 patients will affect CB morphology. The aim of this study is to investigate whether CB hypertrophy is present by CTA examination in patients with COVID-19 pneumonia.

## Materials and Methods

### Study Protocol

After obtaining approval from the hospital ethics committee for the study (No: FSM EAH- 2022-57), a retrospective review of the patients who were treated for COVID-19 pneumonia in our institution in 2020 and 2021 was performed. Patients who had carotid CTA examinations for any reason at least 3 months after the diagnosis of COVID-19 were included in the study. The CTA protocol for cervical vascular structures has been standardized in the existing CT system in our institution. CTAs were performed on a 128 detector (GE Optima CT660 Freedom Edition, General Electric Medical Systems, WI, United States) multislice CT scanner using 0.625 mm slice thickness, 0.984 pitch, 120 kV, automatic mA, and soft tissue reconstruction. Scanning was performed with automatic intravenous injection of iodinated contrast agent (Opaxol, 350 mg/mL) at a dose of 1–1.2 mL/kg and an injection rate of 4 mL/s. Scanning was initiated with an ROI located at the level of the aortic arch, with automatic triggering when values ≥120 Hounsfield Units were reached following a loading bolus. Three planes reformat images were created from the source images. Image processing and postprocessing were performed on the GE Advantage Workstation (GE Healthcare, Buc, France). Measurements were made using axial and reformat images.

### Study Population and Demographics

Demographic data such as age and gender, obtained from the electronic medical records of our hospital, were recorded for all subjects. Among the patients who were treated for COVID-19 pneumonia and had CTA examination, included in the study, the time between diagnosis and CTA was at least 3 months and at most 11 months. We found 189 patients who were treated for COVID-19 pneumonia and had CTA examinations at least 3 months later. The presence of chronic diseases such as hypertension, congestive heart failure, asthma, chronic obstructive pulmonary disease, and cirrhosis was investigated in the hospital records of these patients, and 63 subjects with these diseases were excluded from the study.

**FIGURE 1:**
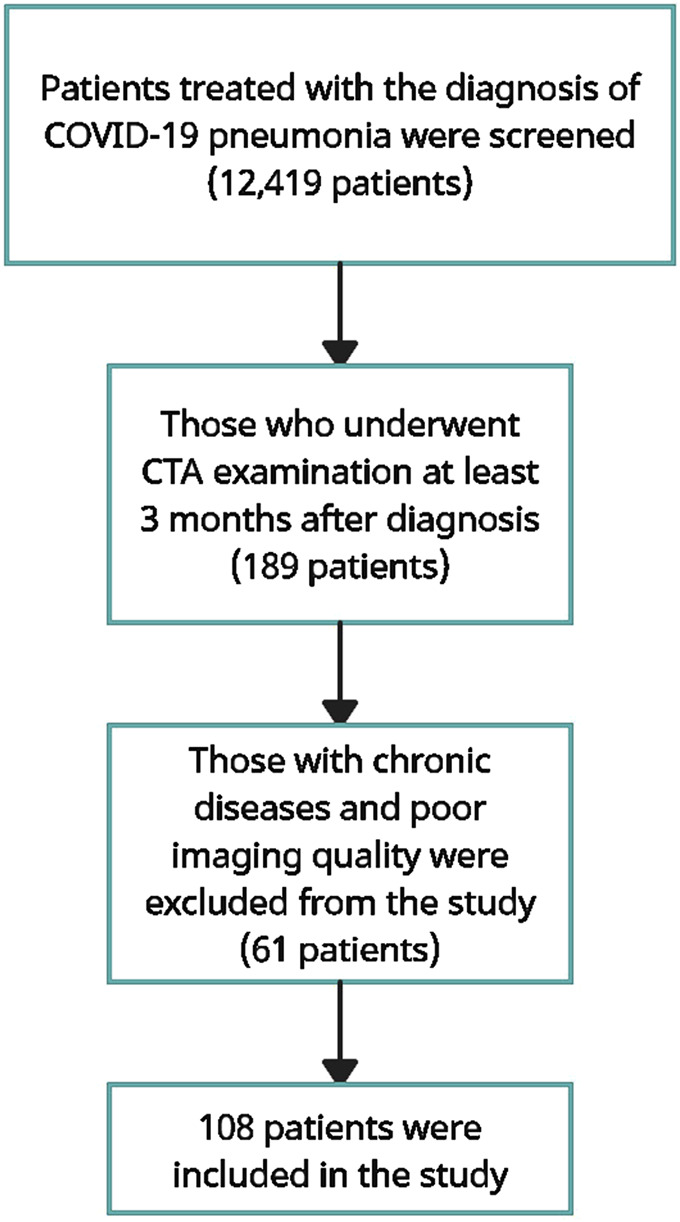
Flow diagram describing the selection of cases.

Then, CB measurements of all subjects were started. Area measurements were performed with the ROI made in a way that the CBs were in contact with their outer edges all around at the cross-section where they appeared the widest. If the same subject had repeated CTAs, a later examination was used for measurement. Eighteen subjects who did not allow healthy measurements due to patient movements or other reasons or whose carotid body could not be shown were excluded from the study (Figure 1). In selecting the control group, criteria similar to those used in COVID-19 patients were used. Records of patients who underwent CTA examination for various reasons were reviewed. If these patients had chronic diseases such as hypertension, congestive heart failure, asthma, chronic obstructive pulmonary disease, and cirrhosis in their records, they were excluded from the study. In addition, if there was any suspicious information about COVID-19 or any infectious disease record after the COVID-19 pandemic in the patient’s medical records, these subjects were not included in the study. One hundred and four subjects who met these conditions were found and included in the study as the control group.

**FIGURE 2:**
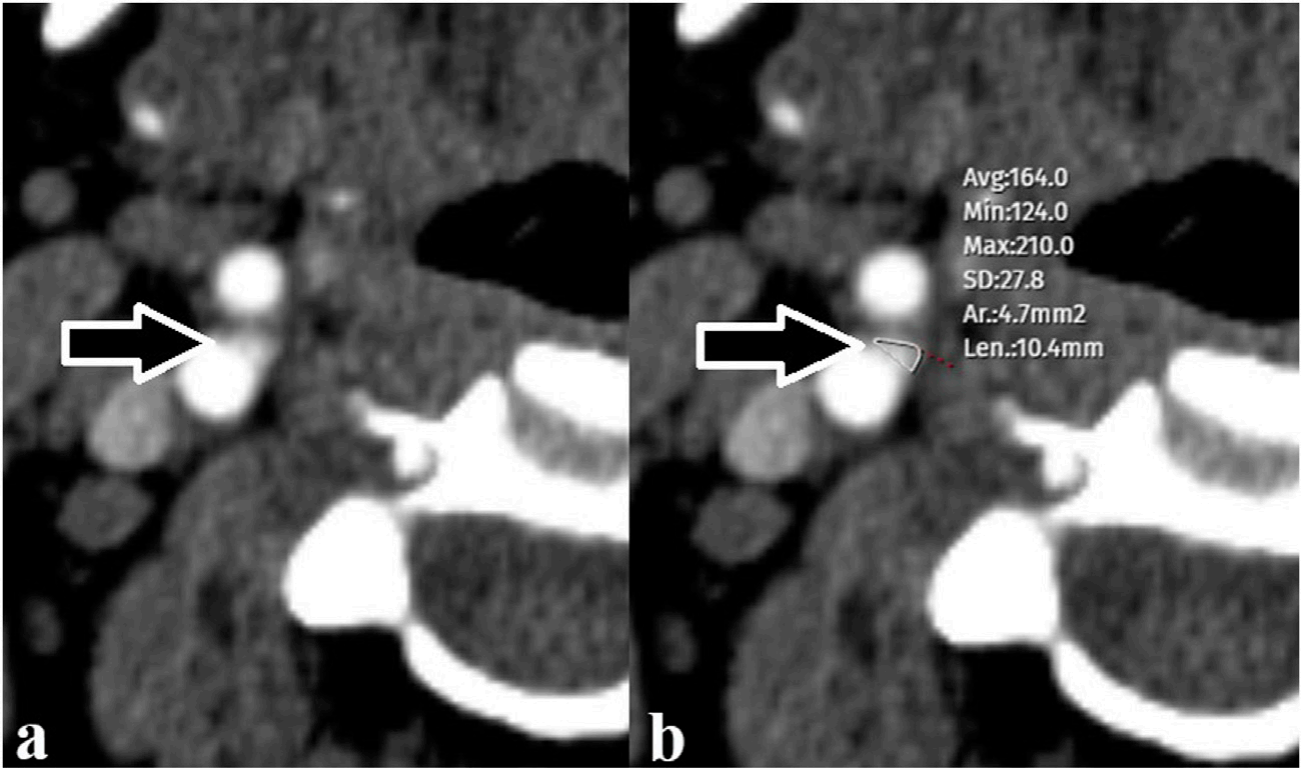
**(A)** Carotid body (arrow) observed at the level of the right carotid bifurcation **(B)** The carotid body (arrow) was measured with a free region of interest (maximum and minimum values in the selected area as Hounsfield Units, mean and standard deviation, area measurement, and the longest dimension of the selection).

The CB appears on CTA as an ovoid enhancing structure at the carotid bifurcation. Measurements were performed at a workstation at 200% magnification and using an consistent window/level (window 600 HU; level 100 HU) by a radiologist with 18 years of experience. In the section where the CB appeared largest in the axial plane, a ROI was drawn tangential to the outer borders of the contrast enhanced CB in order to obtain maximum dimensions. The precision of the electronic caliper that allowed the ROI to be drawn was one tenth of a millimeter. These methods recorded the size of all CBs as areas. The radiologist who made the measurements was blind to the patient’s medical history. CB measurements were performed on the axial view, where the largest dimension was observed (Figure 2).

### Statistical Analysis

Statistical evaluation was performed using IBM SPSS Statistics (version 22, IBM, United States). Data were described using descriptive statistical methods. Median and interquartile range were indicated, as appropriate. The results were evaluated statistically and analyzed using the Mann-Whitney U test as they did not show a normal distribution. All p values were reported in an open form and p < 0.05 was chosen as the level of significance.

## Results

Twelve thousand four hundred nineteen patients who were followed up and treated in our hospital with the diagnosis of COVID-19 pneumonia were examined. The medical records of these patients were scanned and those who had carotid CTA examination at least 3 months after the diagnosis of pneumonia were identified. Those with chronic diseases that may be associated with carotid body size and those whose image quality was not suitable for measurement were excluded from the study, and the remaining 108 patients were included in the study. Similarly, 104 subjects who had carotid CTA examination for any reason and had no chronic disease were considered as the control group (Figure 1).

Of the COVID-19 patients (n = 108), 56 (52%) were female, 52 (48%) were male, 59 (57%) of the control group (n = 104) were female, 45 (43%) was male. The mean age of the COVID-19 patients was 66.5 ± 14.9, while the mean age of the control group was 69.2 ± 15.4.

**FIGURE 3:**
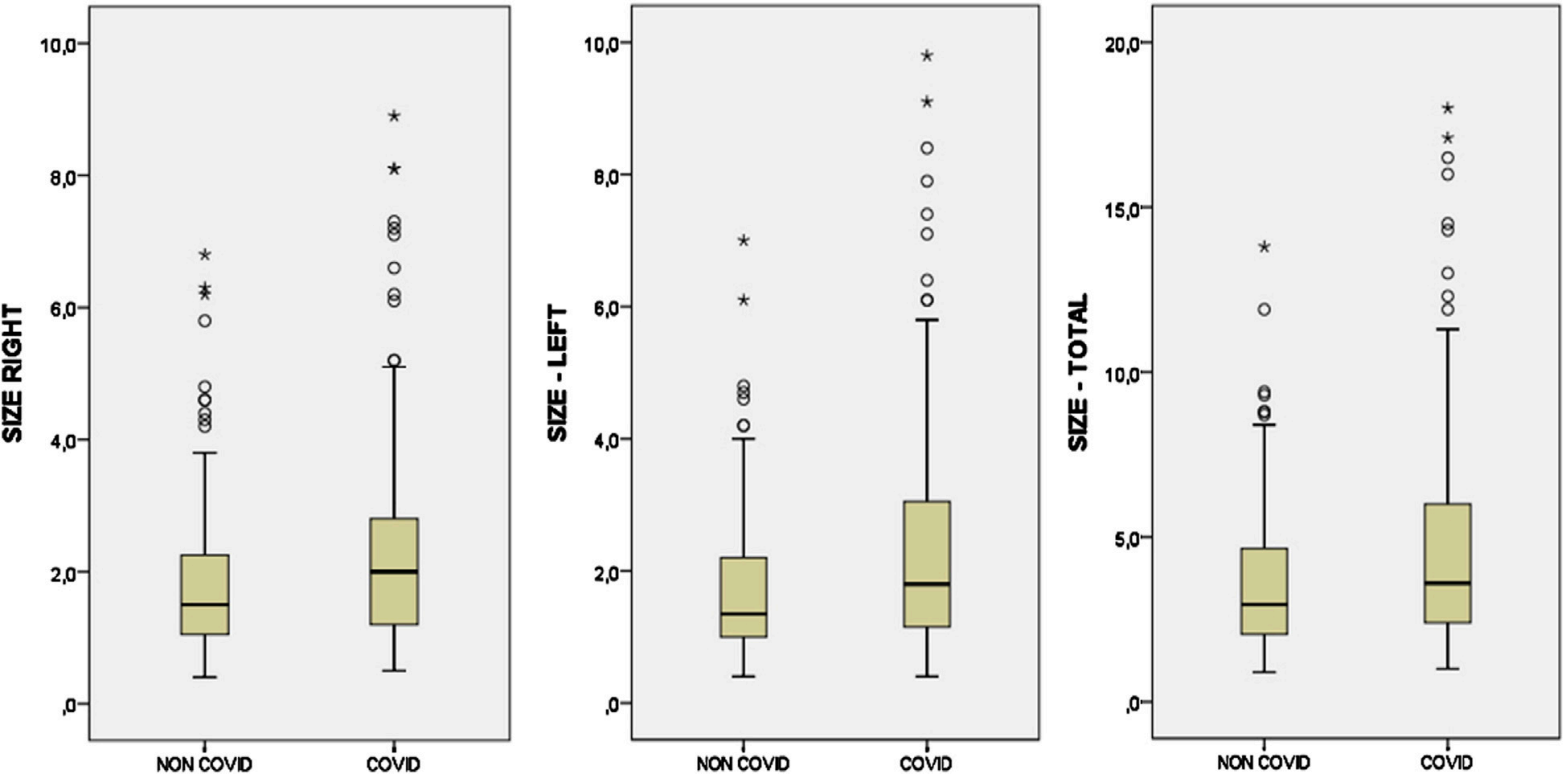
Box plots showing the comparison of right, left, and total carotid body sizes in COVID and control (NON-COVID) groups.

In COVID-19 patients, the difference between the right CB size (median = 2) and the left CB size (median = 1.8) was not statistically significant. Similarly, no statistically significant difference was found between the right CB size (median = 1.5) and left CB size (median = 1.35) in the control group. While the median value of the right and left total CB size (mm2) in the COVID-19 patient group was 3.6, the median value of the total CB size in the control group was 2.95 (Figure 3). There was a statistically significant difference between the two groups and the total CB size was significantly larger in patients with COVID-19 pneumonia (U = 4,409.50, z = −2.70, p = 0.007, r = −18.54) (Table 1). The total CB size in the COVID-19 patient group was approximately 30%–35% higher than in the control group. The median values of total CB size were 3.60 in male subjects and 3.10 in female subjects. CB size was higher (15%–20%) in male subjects than in female subjects, although it was not statistically significant (p = 0.054).

**TABLE 1:** Carotid body size (mm^2^).

	Median	Interquartile range	Mean rank	Sum of ranks	U	P
Control	2.95	2.7	94.90	9,869.50	4,409.50	0.007
COVID-19	3.6	3.7	117.67	12,708.50		

## Discussion

After a significant period with SARS-CoV-2 infection and the COVID-19 epidemic, the ongoing long-term effects of the disease continue to be discussed on the agenda. Some answers regarding the pathophysiology experienced by COVID-19 patients, which may explain some of the symptoms and outcomes in COVID-19 patients, continue to be sought. It is thought that the physiological and pathophysiological responses involving peripheral chemoreceptors that may occur in patients infected with the SARS-CoV-2 virus may be useful in explaining the course and long-term effects of the disease [27].

Given the low partial pressure of oxygen (PaO2) in the arterial blood of patients infected with the SARS-CoV-2 virus [28, 29], the carotid bodies and the resulting reflex responses likely play a central role in symptoms and outcomes [30, 31]. It should be noted that the response to hypoxia may vary from person to person due to different thresholds of oxygen partial pressure required to activate the carotid body [32]. In addition, it should not be forgotten that the multisystemic effects of COVID-19 disease may cause differences in some of the body’s responses [33]. We need to recognize that each COVID-19 patient has an unpredictable combination of multiple factors that determine the course of the infection and the symptoms experienced. Therefore, such factors may alter an individual’s response profile to hypoxia and the degree to which carotid body activation is affected in COVID-19 patients. Carotid bodies are known to be not only sensors of blood gases, but also multifunctional arterial sensors that respond to inflammation, low blood pressure, sympathetic activity, and various metabolically related hormones. Therefore, the involvement of carotid bodies in COVID-19 patients may not be limited only to the response to hypoxia [30, 32, 33].

Additionally, it is reported that SARS-CoV-2 can circulate in the blood and infect ACE2-expressing tissues such as lung epithelium, olfactory neuroepithelium, cardiovascular system, and gastrointestinal tract, thus causing multiple and different functional changes [26]. Based on this, it is stated that the SARS-CoV-2 virus can directly cause CB infection and may be the cause of the differences in the degree of hypoxia and different responses to hypoxia observed in COVID-19 patients [25]. It is very difficult to demonstrate possible CB involvement in COVID-19 patients. Since it is not possible to get results with a biopsy, other researchers recommend examining CBs in autopsy studies [25]. In our study, the fact that the change in the size of the CBs was revealed by CTA suggests that radiological ideas can be obtained about the possible involvement of the CBs. Of course, follow-up studies and correlation with autopsy results are required to determine whether the damage is temporary or not.

Due to its high vascularity, normal CBs can be visualized in routine CTA examinations [34]. In previous studies, it has been reported that CB hypertrophy (physical size or weight) can be seen in various chronic diseases such as hypertension, congestive heart failure, asthma, chronic obstructive pulmonary disease and cirrhosis [12–17]. In our study, a statistically significant enlargement of approximately 30%–35% areal in CB dimensions was shown in patients who had had COVID-19 pneumonia at least 3 months ago, compared to the control group. This expansion rate appears to be similar to previous hypertension-related [3] and DM-related [35] CB growth rates. The first implication from these results is that CB expansion, which may be associated with COVID-19 or comorbidities leading to increased sympathetic activity, is limited. In these cases, there is not enough growth to mimic a CB paraganglioma.

The correlation between CB size and cardiovascular disease has long been recognized [12, 13, 15–17]. In addition, recent studies show that there is a relationship between the presence and progression of comorbidities associated with increased sympathetic activity, especially CHF and HT, and the size of CB [36]. In addition, it has been reported that CB size and activity may be associated with mortality in CHF patients [37, 38]. When there is an increase in CB activity, it is thought that there is an increase in glomus cell mass and fibrosis, thus increasing the size of CB [23]. However, the mechanisms underlying CB expansion are not fully known. In cases where there is increased sympathetic activity, an increase in the amount of angiotensin II and downregulation of nitric oxide in the glomus occur, which is said to contribute to enlargement [36, 39, 40]. The autonomic nervous system plays a modulatory role on the immune system and is thought to have a potential role in the complex immunological state seen in COVID-19 patients. Sympathetic nerve fibers innervate most lymphoid organs, including bone marrow [41], and many different immune cell types have adrenergic receptors [42]. The effects of the sympathetic system on the immune system are quite complex, and there is evidence of a proinflammatory effect in certain tissues, experimental or pathological conditions. Therefore, there are studies showing that the increase in sympathetic activity is proportional to the presence and disease severity in patients with COVID-19 pneumonia [21, 43, 44]. Patients with COVID-19 pneumonia may experience increased sympathetic discharge through respiratory failure, changes in blood gases, immune/inflammatory factors, or angiotensin-converting enzyme (ACE)1/ACE2 imbalance [20, 45, 46]. The increase in CB size in patients with COVID-19 pneumonia supports a possible functional relationship between CB size and increased sympathetic activity.

Although CB size appears to be associated with conditions associated with CB activity and some diseases, the exact causes and mechanism of formation have not been fully elucidated. In addition, on the contrary, a CB that has grown spontaneously and has increased activity may also lead to the presence and progression of the aforementioned diseases. Or, some diseases whose role has not been revealed yet may cause CB hyperactivity, which may lead to the progression of diseases that we think are related. Most likely, all of these scenarios contribute more or less to CB growth [47].

This study has some important limitations. First of all, in the use of 25 cm FOV and 512 × 512 matrix size we use, even a few pixel differences can lead to a size difference, which can be considered significant, considering the limitation of the monitor resolution. That is, the detected 30%–35% CB growth, although statistically significant, is not very precise for the measurement method used. A few pixels selected or not selected by the measuring reader can change the result. Therefore, in a large sample group, the enlargement can be detected statistically and evaluated as significant. However, it can be difficult to decide whether growth is present or absent in a single CTA. In addition, in terms of the reliability of the measurements, one of the limitations of the study is that measurements were made by more than one radiologist and interobserver consistency was not evaluated. Since our study was conducted retrospectively and our data were limited to hospital records, we could not include potential confounding variables and specific variables that could affect CB size in the evaluation. Additionally, due to deficiencies in the data, the vaccination status of the patients and the medication they received in the treatment of COVID-19 were not taken into account in the evaluation.

In our study, for the CB size, the area measurement obtained with the ROI drawn in such a way that the contact around the CB was not interrupted was used in the axial view, where the organ appears the widest. However, the CB organ is often not spherical but may have lobulated contours. Therefore, the area measurement we use may not always correlate perfectly with the volume of the CB. Although it is possible to measure volume with CTA, it is technically difficult to measure volume using CTA due to the small size of CB. However, it has been reported in previous studies that diameter measurement with CTA in the detection of CB growth is compatible with data obtained by anatomical dissection [3]. However, in another study, it was reported that the diameter measurement made with CTA showed greater growth, and it is not possible to compare the measurement difference with CTA because many anatomical studies report CB size only in terms of weight or volume [14–17, 34].

In this retrospective study, we aimed to evaluate the CB size in CTA examinations performed for various reasons in patients with COVID-19 pneumonia at least 3 months after the diagnosis of pneumonia. However, CTA reviews were routine reviews that were not optimized for CB viewing. Therefore, image quality can be considered as optimal or poor for evaluating CB size. The reasons for this are the lack of applications such as small FOV for CB location localization, high resolution and special adjustment of bolus timing, and the use of a routine examination protocol. In future studies, results with high measurement precision can be obtained with specially adjusted protocols for CB imaging.

In addition, although comorbidities are routinely questioned in patients treated with a diagnosis of COVID-19 pneumonia, the study was conducted retrospectively using hospital records. Therefore, there may be insufficient hospital records regarding the presence of chronic diseases that may lead to an increase in CB size. Similarly, hospital records were used in the control group, and it is not possible to say that chronic diseases such as hypertension, congestive heart failure, asthma, chronic obstructive pulmonary disease and cirrhosis, as well as conditions such as smoking and sleep apnea were excluded definitively. Subjects were excluded from the study in the presence of the above-mentioned diseases in both patients with COVID-19 and the control group, regardless of the severity and duration of the disease. The time interval for CTA examination after diagnosis of COVID-19 pneumonia was 3–11 months, longer follow-ups will provide valuable information in future studies.

## Conclusion

It is possible for CB to be affected directly or indirectly in COVID-19 patients. No method has been described that can directly indicate possible CB retention for COVID-19 patients. Although our study is a retrospective study conducted with a limited sample and has some limitations, our findings show that CB size can be determined by CTA examination. In our study, CB sizes were found to be statistically significantly larger in patients with COVID-19 pneumonia than in the control group. This study appears to be the first radiological study in the literature investigating the possible relationship between COVID-19 pneumonia and an increase in CB size. Additional studies are needed that include longer follow-ups after COVID-19 pneumonia and take into account other diseases and factors that may cause an increase in CB sizes according to their severity and duration. Also, future studies based on volume measurement of CBs using better protocols customized for CB imaging in CTA examinations will provide further and useful information.

## Summary Table

What is Known About This Topic?

• Many pathophysiological theories are discussed regarding the increase in sympathetic activity with respiratory failure in COVID-19 patients.

What This Work Adds

• In patients who have had COVID-19 pneumonia, the size of the carotid bodies is statistically significantly increased by 30%–35% compared to the control group.
• This work represents an advance in biomedical science because the findings reveal that CB sizes increase in COVID patients, and this finding offers a new perspective on COVID disease.

## Data Availability

The raw data supporting the conclusions of this article will be made available by the authors, without undue reservation.

## References

[1] Ballering AV van Zon SKR Hartman TCO Rosmalen JGM, Lifelines Corona Research Initiative. Persistence of Somatic Symptoms after COVID-19 in the Netherlands: An Observational Cohort Study. Lancet (2022) 400:452–61. 10.1016/s0140-6736(22)01214-4 35934007 PMC9352274

[2] Mao L Jin H Wang M Hu Y Chen S He Q Neurologic Manifestations of Hospitalized Patients with Coronavirus Disease 2019 in Wuhan, China. JAMA Neurol (2020) 77:683–90. 10.1001/jamaneurol.2020.1127 32275288 PMC7149362

[3] Siemieniuk RA Bartoszko JJ Zeraatkar D Kum E Qasim A Martinez JPD Drug Treatments for Covid-19: Living Systematic Review and Network Meta-Analysis. BMJ (2020) 370:m2980. 10.1136/bmj.m2980 32732190 PMC7390912

[4] Bull-Otterson L Baca S Saydah S Boehmer TK Adjei S Gray S Post–COVID Conditions Among Adult COVID-19 Survivors Aged 18–64 and ≥65 Years — United States, March 2020–November 2021. MMWR Morb Mortal Wkly Rep (2022) 71:713–7. 10.15585/mmwr.mm7121e1

[5] Ceban F Ling S Lui LM Lee Y Gill H Teopiz KM Fatigue and Cognitive Impairment in Post-COVID-19 Syndrome: A Systematic Review and Meta-Analysis. Brain Behav Immun (2022) 101:93–135. 10.1016/j.bbi.2021.12.020 34973396 PMC8715665

[6] Al-Aly Z Bowe B Xie Y. Long COVID after Breakthrough SARS-CoV-2 Infection. Nat Med (2022) 28:1461–7. 10.1038/s41591-022-01840-0 35614233 PMC9307472

[7] Ayoubkhani D Bosworth ML King S Pouwels KB Glickman M Nafilyan V Risk of Long Covid in People Infected with SARS-CoV-2 after Two Doses of a COVID-19 Vaccine: Community-Based, Matched Cohort Study. Open Forum Infect Dis (2022) 12:465. 10.1093/ofid/ofac464 PMC9494414 36168555

[8] Davis HE McCorkell L Vogel JM Topol EJ. Long COVID: Major Findings, Mechanisms and Recommendations. Nat Rev Microbiol (2023) 21(3):133–46. 10.1038/s41579-023-00896-0 36639608 PMC9839201

[9] Larsen NW Stiles LE Shaik R Schneider L Muppidi S Tsui CT Characterization of Autonomic Symptom Burden in Long COVID: A Global Survey of 2,314 Adults. Front Neurol (2022) 19:1012668. 10.3389/fneur.2022.1012668 PMC9639503 36353127

[10] Bach K. Is ‘long Covid’ Worsening the Labor Shortage? (2022). Available online at: https://www.brookings.edu/research/is-long-covid-worsening-the-labor-shortage/ (Accessed on April 01, 2024).

[11] Eyzaguirre C Zapata P. Perspectives in Carotid Body Research. J Appl Physiol (1984) 57:931–57. 10.1152/jappl.1984.57.4.931 6150019

[12] Kato K Wakai J Matsuda H Kusakabe T Yamamoto Y. Increased Total Volume and Dopamine β-hydroxylase Immunoreactivity of Carotid Body in Spontaneously Hypertensive Rats. Auton Neurosci (2012) 169:49–55. 10.1016/j.autneu.2012.03.005 22546625

[13] Habeck JO. Peripheral Arterial Chemoreceptors and Hypertension. J Autonom Nerv Sys (1991) 34:1–7. 10.1016/0165-1838(91)90003-l 1940013

[14] Habeck JO. Morphological Findings at the Carotid Bodies of Humans Suffering from Different Types of Systemic Hypertension or Severe Lung Diseases. Anat Anz (1986) 162:17–27. 3752531

[15] Edwards C Heath D Harris P. The Carotid Body in Emphysema and Left Ventricular Hypertrophy. J Pathol (1971) 104:1–13. 10.1002/path.1711040102 4254957

[16] Bencini C Pulera N. The Carotid Bodies in Bronchial Asthma. Histopathol (1991) 18:195–200. 10.1111/j.1365-2559.1991.tb00826.x 2045071

[17] Heath D Edwards C Harris P. Post-Mortem Size and Structure of the Human Carotid Body. Thorax (1970) 25:129–40. 10.1136/thx.25.2.129 4245588 PMC472136

[18] Carnagarin R Lambert GW Kiuchi MG Nolde JM Matthews VB Eikelis N Effects of Sympathetic Modulation in Metabolic Disease. Ann N Y Acad Sci (2019) 1454:80–9. 10.1111/nyas.14217 31424101

[19] Díaz HS Toledo C Andrade DC Marcus NJ Del Rio R. Neuroinflammation in Heart Failure, New Insights for an Old Disease. J Physiol (2020) 598:33–59. 10.1113/JP278864 31671478

[20] Gurwitz D. Angiotensin Receptor Blockers as Tentative SARS-CoV-2 Therapeutics. Drug Dev Res (2020) 81:537–40. 10.1002/ddr.21656 32129518 PMC7228359

[21] Porzionato A Emmi A Barbon S Boscolo-Berto R Stecco C Stocco E Sympathetic Activation, A Potential Link between Comorbidities and COVID-19. FEBS J (2020) 287:3681–8. 10.1111/febs.15481 32779891 PMC7405290

[22] Zhang H Penninger JM Li Y Zhong N Slutsky AS. Angiotensin-converting Enzyme 2 (ACE2) as a SARS-CoV-2 Receptor, Molecular Mechanisms and Potential Therapeutic Target. Intensive Care Med (2020) 46:586–90. 10.1007/s00134-020-05985-9 32125455 PMC7079879

[23] Shouman K Vanichkachorn G Cheshire WP Suarez MD Shelly S Lamotte GJ Autonomic Dysfunction Following COVID-19 Infection, an Early Experience. Clin Auton Res (2021) 31:385–94. 10.1007/s10286-021-00803-8 33860871 PMC8050227

[24] Gordon DE Jang GM Bouhaddou M Xu J Obernier K White KM A SARS-CoV-2 Protein Interaction Map Reveals Targets for Drug Repurposing. Nature (2020) 583:459–68. 10.1038/s41586-020-2286-9 32353859 PMC7431030

[25] Villadiego J Ramírez-Lorca R Cala F Labandeira-García JL Esteban M Toledo-Aral JJ Is Carotid Body Infection Responsible for Silent Hypoxemia in COVID-19 Patients? Function (2021) 2:zqaa032. 10.1093/function/zqaa032 35321097 PMC7717325

[26] Li H Liu L Zhang D Xu J Dai H Tang N SARS-CoV-2 and Viral Sepsis: Observations and Hypotheses. Lancet. (2020) 395:1517–20. 10.1016/S0140-6736(20)30920-X 32311318 PMC7164875

[27] Machado BH Paton JFR. Relevance of Carotid Bodies in COVID-19: A Hypothetical Viewpoint. Auton Neurosci (2021) 233:102810. 10.1016/j.autneu.2021.102810 33894532 PMC8052558

[28] Sartini C Tresoldi M Scarpellini P Tettamanti A Carcò F Landoni G Respiratory Parameters in Patients with COVID-19 after Using Noninvasive Ventilation in the Prone Position outside the Intensive Care Unit. JAMA (2020) 323:2338–40. 10.1001/jama.2020.7861 32412606 PMC7229533

[29] Chen T Wu D Chen H Yan W Yang D Chen G Clinical Characteristics of 113 Deceased Patients with Coronavirus Disease 2019: Retrospective Study. BMJ (2020) 368:m1091. 10.1136/bmj.m1091 32217556 PMC7190011

[30] Porzionato A Emmi A Stocco E Barbon S Boscolo-Berto R Macchi V The Potential Role of the Carotid Body in COVID-19. Am J Physiol Lung Cell Mol Physiol (2020) 319:L620–L626. 10.1152/ajplung.00309.2020 32755325 PMC7516384

[31] Holmes AP. Damage Control: Carotid Body Activation and Remodelling in Response to Aseptic Tissue Injury. Exp Physiol (2020) 105(9):1467–9. 10.1113/EP088923 32735375

[32] Tobin MJ Laghi F Jubran A. Why COVID-19 Silent Hypoxemia Is Baffling to Physicians. Am J Respir Crit Care Med (2020) 302(3):356–60. 10.1164/rccm.202006-2157CP PMC7397783 32539537

[33] Gattinoni L Chiumello D Caironi P Busana M Romitti F Brazzi L COVID-19 Pneumonia: Different Respiratory Treatments for Different Phenotypes? Intensive Care Med (2020) 46:1099–102. 10.1007/s00134-020-06033-2 32291463 PMC7154064

[34] Nguyen RP Shah LM Quigley EP Harnsberger HR Wiggins RH. Carotid Body Detection on CT Angiography. AJNR Am J Neuroradiol (2011) 32:1096–9. 10.3174/ajnr.A2429 21393408 PMC8013152

[35] Cramer JA Wiggins RH Fudim M Engelman ZJ Sobotka PA Shah LM. Carotid Body Size on CTA, Correlation with Comorbidities. Clin Radiol (2014) 69:33–6. 10.1016/j.crad.2013.08.016 24156799

[36] Paton JFR Sobotka PA Fudim M Engelman ZJ Hart ECJ McBryde FD The Carotid Body as a Therapeutic Target for the Treatment of Sympathetically Mediated Diseases. Hypertension (2013) 61:5–13. 10.1161/HYPERTENSIONAHA.111.00064 23172927

[37] Ponikowski P Chua TP Anker SD Francis DP Doehner W Banasiak W Peripheral Chemoreceptor Hypersensitivity, an Ominous Sign in Patients with Chronic Heart Failure. Circulation (2001) 104:544–9. 10.1161/hc3101.093699 11479251

[38] Giannoni A Emdin M Bramanti F Iudice G Francis DP Barsotti A Combined Increased Chemosensitivity to Hypoxia and Hypercapnia as a Prognosticator in Heart Failure. J Am Coll Cardiol (2009) 53:1975–80. 10.1016/j.jacc.2009.02.030 19460611

[39] Prabhakar NR Semenza GL. Gaseous Messengers in Oxygen Sensing. J Mol Med (2012) 90:265–72. 10.1007/s00109-012-0876-1 22349394

[40] Tan Z-Y Lu Y Whiteis CA Simms AE Paton JFR Chapleau MW Chemoreceptor Hypersensitivity, Sympathetic Excitation, and Overexpression of ASIC and TASK Channels before the Onset of Hypertension in SHR. Circ Res (2010) 106:536–45. 10.1161/CIRCRESAHA.109.206946 20019330 PMC2846115

[41] Felten DL Felten SY Carlson SL Olschowka JA Livnat S. Noradrenergic and Peptidergic Innervation of Lymphoid Tissue. J Immunol (1985) 135:755–65. 10.4049/jimmunol.135.2.755 2861231

[42] Brodde OE Engel G Hoyer D Bock KD Weber F. The Beta-Adrenergic Receptor in Human Lymphocytes: Subclassification by the Use of a New Radio-Ligand, (+/-)-125 Iodocyanopindolol. Life Sci (1981) 29:2189–98. 10.1016/0024-3205(81)90490-2 6275222

[43] Li YC Bai WZ Hashikawa T. The Neuroinvasive Potential of SARS-CoV2 May Play a Role in the Respiratory Failure of COVID-19 Patients. J Med Virol (2020) 92:552–5. 10.1002/jmv.25728 32104915 PMC7228394

[44] Pedersen SF Ho YC. SARS-CoV-2, a Storm Is Raging. J Clin Invest (2020) 130:2202–5. 10.1172/JCI137647 32217834 PMC7190904

[45] Jammoul M Naddour J Madi A Reslan MA Hatoum F Zeineddine J Investigating the Possible Mechanisms of Autonomic Dysfunction Post-COVID-19. Auton Neurosci (2023) 245:103071. 10.1016/j.autneu.2022.103071 36580747 PMC9789535

[46] Fedorowski A Fanciulli A Raj SR Sheldon R Shibao CA Sutton R. Cardiovascular Autonomic Dysfunction in Post-COVID-19 Syndrome: A Major Health-Care Burden. Nat Rev Cardiol (2024) 1:17. 10.1038/s41569-023-00962-3 38163814

[47] Kumar P. The Carotid Body in Cardiovascular Disease, More Chicken and Egg Than Horse and Cart? J Physiol (Lond) (2012) 590:4123. 10.1113/jphysiol.2012.239921 22962032 PMC3473269

